# Advances in biotechnology and genomics of switchgrass

**DOI:** 10.1186/1754-6834-6-77

**Published:** 2013-05-12

**Authors:** Madhugiri Nageswara-Rao, Jaya R Soneji, Charles Kwit, C Neal Stewart

**Affiliations:** 1 Department of Plant Sciences, The University of Tennessee, 252 Ellington Plant Sciences, 2431 Joe Johnson Dr., Knoxville, TN 37996, USA; 2 Department of Biological Sciences, Polk State College, Winter Haven, FL 33881, USA; 3 BioEnergy Science Center, Oak Ridge National Laboratory, Oak Ridge, TN 37831, USA

**Keywords:** Biofuels, Expressed sequence tags, Genetic engineering, Genome sequencing, Lignin biosynthesis, microRNAs, Molecular markers

## Abstract

Switchgrass (*Panicum virgatum* L.) is a C_4 _perennial warm season grass indigenous to the North American tallgrass prairie. A number of its natural and agronomic traits, including adaptation to a wide geographical distribution, low nutrient requirements and production costs, high water use efficiency, high biomass potential, ease of harvesting, and potential for carbon storage, make it an attractive dedicated biomass crop for biofuel production. We believe that genetic improvements using biotechnology will be important to realize the potential of the biomass and biofuel-related uses of switchgrass. Tissue culture techniques aimed at rapid propagation of switchgrass and genetic transformation protocols have been developed. Rapid progress in genome sequencing and bioinformatics has provided efficient strategies to identify, tag, clone and manipulate many economically-important genes, including those related to higher biomass, saccharification efficiency, and lignin biosynthesis. Application of the best genetic tools should render improved switchgrass that will be more economically and environmentally sustainable as a lignocellulosic bioenergy feedstock.

## Introduction

**Figure 1:**
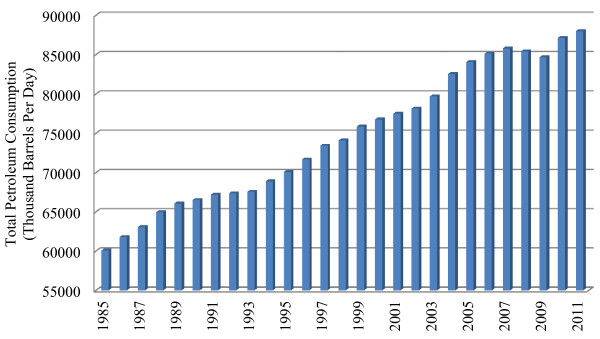
Total world petroleum consumption (thousand barrels/day) [Source: U.S. Energy Information Administration (EIA)].

Resource consumption by humans continues to proceed at arguably unsustainable levels. In recent times, worldwide consumption of non-renewable fossil fuel reserves has increased drastically (U.S. Energy Information Administration; http://www.eia.gov/; Figure 1). With high rates of consumption anticipated and an ever-increasing population, a great challenge will be meeting the growing demand for energy for transportation, heating and industrial processes, and providing the raw industrial materials in a sustainable way [1]. Fossil fuels supply more than 80% of energy consumed globally and contribute to atmospheric greenhouse gases, declining water tables and climate change [2,3]. All of these factors naturally lead to the development of renewable energy sources.

Biomass and biomass-derived fuels may be able to provide a partial solution to today’s energy challenges. In the last decade, there has been increased interest in dedicated biomass crops for biofuels [4]. It was considered that bioenergy provided by starch, sugar, and oils from plants would be crucial for accomplishing the goals of incremental substitution of petroleum-based transportation fuels in addition to reducing CO_2 _emissions [5,6]. However, first-generation biofuels were produced from traditional food and feed crops (e.g., sugarcane, corn, sugar beet), which may lead to supply shortages, and, in turn, to an increase in food prices [3,7].

Even though plant-derived biofuels are renewable and, for the most part, carbon-neutral, they have been condemned for being associated with the loss of biological diversity and unfavorable consequences of changes in land use patterns [5]. These shortcomings led to a vision of developing second-generation lignocellulosic bioenergy crops, wherein stems, leaves, and/or husks of plants such as switchgrass, *Miscanthus*, jatropha, and poplar, may be used for the production of biofuels. In contrast to the easily-processed sugars and oils of first-generation bioenergy feedstocks, lignocellulosic biomass contains hard-to-digest matter from cell walls of grasses, crop residue, and woody biomass. One goal for the selection of second-generation bioenergy crops is that they should be able to grow on ‘marginal’ and low-cost land not suited for food crops, thus removing competition between the uses of land for food or fuel production [8]. Challenges remain in making second-generation bioenergy crops a reality. Among these are: (a) how to sustainably maximize the yield per hectare of biomass while minimizing agricultural inputs, (b) how to truly avoid competition with food and feed production, (c) how to increase the efficiency of biomass digestion by microbes and other processes [9], and (d) whether transgenic plants can be used [10].

**Figure 2:**
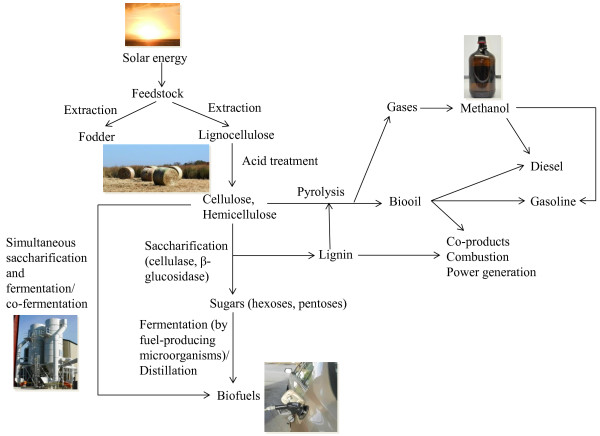
Flow chart of biofuel production in switchgrass [Photo credits: M Nageswara-Rao].

Among all potential second-generation bioenergy crops, switchgrass (*Panicum virgatum* L.) has received, perhaps, the most attention as a dedicated lignocellulosic biofuel crop, beginning in the 1980s [11,12] (Figure 2). Switchgrass is a member of the Paniceae tribe of grasses and belongs to the family Poaceae. It is native to North America and widely adapted; growing from 20°-60° north latitude and east of 100° west longitude [13,14]. It exhibits tremendous diversity in its form and has been categorized into two ecotypes: upland and lowland [15,16]. It can be grown on lands less-suitable for traditional agricultural crops for the production of biofuels, such as ethanol and butanol, from cellulose [17]. Switchgrass readily thrives on marginal land as a result of its deep-rooting habit, C_4_ photosynthetic metabolism, among other traits [13]. Its perennial growth habit, wide adaptation, excellent conservation attributes, compatibility with conventional farming practices, ease of harvesting, handling, storage and amenability for being handled and stored both as wet or dry feedstock has made it a popular choice for biofuel feedstock crop [18-20]. Its high yielding potential on marginal lands and high yields across much of the eastern United States, especially the mid-South has set it apart from most other biofuel alternatives [12,21,22]. Switchgrass yields higher net energy than required to cultivate, harvest and convert it into cellulosic ethanol leading to much improved greenhouse gas balance compared with gasoline [23].

The importance of switchgrass as a bioenergy feedstock has increased interest in the generation of new cultivars optimized for energy production through breeding, biotechnology and management research. Improvement of biomass yield and nutritional quality should be amenable by conventional breeding. However, drastically better conversion of cell walls into fuels might not be possible by conventional breeding; genomics, biotechnology, systems- and synthetic biology tools might be required. Genomics and systems biology allow the identification and characterization of key genes that underlie critical fundamental processes. Overexpression of novel genes or knockdown of the expression of key endogenous genes can alter cell walls to dramatically improve fuel yield of switchgrass. The present scenario and the future prospects of the utilization of molecular and biotechnological tools for the genetic improvement of switchgrass have been emphasized in this review. While it is beyond the scope of this review, we envision that advanced biotechnology tools and synthetic biology will likely be required to optimize desired genetic improvements.

## Biotechnological tools for genetic improvement

### Tissue culture

Efficient switchgrass cell and tissue culture is required for the production of transgenic plants as well as vegetative propagation. Prior to 1991, little switchgrass tissue culture research had been conducted. The initiation of US Bioenergy Feedstock Development Program enhanced opportunities for the long-term improvement of switchgrass [11]. Thus, in the 1990s, this program spurred research exploring explant types, tissue culture and regeneration of switchgrass with the ultimate goal of increasing the resource-base for developing transgenic lines. Switchgrass is amenable to regeneration after somatic embryogenesis and organogenesis.

### Embryogenic callus

Somatic embryogenesis was used by Denchev and Conger [24] who reported high frequency plantlet regeneration. They used mature caryopses (seeds) and young leaf segments of the lowland cultivar ‘Alamo’ as explants to produce embryogenic callus on solidified Murashige and Skoog (MS) medium containing 2,4-dichlorophenoxyacetic acid (2,4-D) and 6-benzylaminopurine (BAP). The ease of handling and callus induction from mature caryopses made these valuable explants. When leaves were used as explants, there was a response gradient with regards to tissue age for callus initiation; young tissue is better than old tissue. Although somatic embryogenesis could be induced from embryogenic calli, regeneration of somatic embryos directly from the cells of the explants was not observed [24]. Somatic embryogenesis has also been reported from young infloresences of ‘Alamo’ [25,26]. The cyclic production of plants from embryogenic callus renders this technique a viable option for rapid clonal propagation of switchgrass. However, compared with seed production, clonal propagation would be quite expensive and probably only used for the most valuable lines.

One disadvantage to the use of embryogenic callus- and seed-derived callus systems is that they generally have limited lifespans (months) of usefulness before they cease to be regenerable. Whereas the longevity of embryo viability can be only two months, the recently described switchgrass medium, LP9, increased the viability of callus and the ability to maintain it for a duration of over six months, making it more efficient for use in a transformation pipeline [27]. LP9 combined N_6 _macroelements and B_5 _microelements for the production and maintenance of switchgrass callus and its regeneration [27]. Also, the callus obtained was categorized as type II callus, which is more effective in grass transformation and regeneration [27] than type I callus obtained from previously described tissue culture systems [25,26].

### Cell suspension cultures

Cells divide faster in liquid suspension cultures compared with callus cells grown on solidified medium [28]. For large scale propagation, mutant selection, gene transfer and protoplast isolation, development of embryogenic cell suspension cultures would be advantageous. Cell suspension cultures were first obtained by Bob Conger’s group that used young inflorescences of ‘Alamo’ as explants, which could directly yield embryogenic callus, which could be regenerated into plants [25]. This same group [26] showed that the utilization of osmotic pretreatment had a positive effect on the initiation and induction of somatic embryogenesis from suspension cultures derived from in vitro-cultured inflorescences of ‘Alamo.’ It was also observed that younger cultures gave a higher embryogenic response as compared with older cultures [26]. The HR8 line that was developed from a recurrent tissue culture selection of ‘Alamo’ had a higher seed germination capacity, and germinating seeds gave rise to higher percentage of somatic embryogenic callus [29]. Although this HR8 line, and indeed all improved Conger materials other than ‘Alamo2’ have been lost, the improved germplasm demonstrated very rapid propagation. These sorts of materials would have great use in breeding programs [11].

Cell suspensions are also excellent starting materials for the isolation of protoplasts. Protoplasts are useful in a wide range of applications including cell fusion and genetic manipulation [30]. Recently, Mazarei *et al.* reported protoplast isolation from switchgrass cell suspension cultures established from embryogenic callus [31]. They demonstrated that protoplast isolation efficiency was highly dependent on the type of cell suspension. Currently, our and other research groups are using cell suspension cultures for a variety of biotechnology-to-synthetic biology applications including deciphering the cell wall biology for improvments and high throughput multi-target genetic engineering and screening.

### Organogenesis

Organogenesis illustrates a significant capability of plants to adapt to their altering environment; this process allows organ genesis from undifferentiated cells [32-34]. Switchgrass regeneration from organogenesis has been accomplished [24,35]. Explants include mature caryopses, young leaf segments and young seedling explants and MS medium supplemented with auxins (2,4-D or picloram) and BAP is effective [24,35]. The combination of 2,4-D and BAP induced a high regeneration frequency in both nonembryogenic and embryogenic calli derived from mature caryopses, while induction of shoots from young seedling explants was more effective when picloram was used in combination with BAP [35]. Protocols for high-throughput callus induction by plating whole dehusked caryopses and plant regeneration from new, higher yielding switchgrass cvs. ‘NSL’ and ‘SL93’ have been optimized [36]. Seed pretreatments, such as dehusking with sulfuric acid, chilling for two weeks at 4°C prior to plating, and sterilizing with sodium hypochlorite and ethanol, were found to have significant effect on callus induction and subsequent plant regeneration.

### Micropropagation

As mentioned earlier, vegetative/micropropagation using tissue culture might be useful for valuable germplasm and also for research. Advanced regeneration techniques have been developed for switchgrass. For the efficient multiplication of switchgrass genotypes, micropropagation has been established using nodal explants especially the nodes below the top node [37]. Regardless of their position on the culm, all nodes exhibited shoot induction at a similar rate. It was also reported that 500 plantlets could be regenerated from a single parent plant in 12 weeks [37]. Clonal propagation can be used for scaling up the number of plants obtained from selected cultivars, for controlled pollination studies for use in breeding programs, in genetic transformation experiments, and also as an important explant source for additional in vitro culture initiation.

In switchgrass, the regeneration capacity is highly genotype-dependent [38,39]. The recalcitrance of upland cultivars warranted the development of new efficient regeneration systems. Intact seedlings of both lowland (‘Alamo’) and upland (‘Trailblazer’ and ‘Blackwell’) cultivars exhibited multiple shoot regeneration on MS medium supplemented with various combinations of 2,4-D and thidiazuron (TDZ) [38]. This technique of inducing multiple shoots from intact seedlings was less labor intensive and more rapid, efficient and consistent across genotypes, and the shoots appeared to originate from enlarged shoot apice [38]. Since each caryopsis vary for genotype, owing to self-incompatibility and natural outcrossing that is inherent to switchgrass, this system did not have utility for clonal propagation.

Immature inflorescences are a significant resource for *in vitro* culture establishment. Young inflorescences of switchgrass have been utilized for callus induction and plant regeneration [40]. To reduce the damage caused by harvesting, endogenous or exogenous fungal and bacterial contamination, and toxicity of sterilization solutions on inflorescences, growth establishment in axenic cultures might be beneficial. A protocol for *in vitro* production of inflorescences from node cultures derived from greenhouse grown tillers of ‘Alamo’ has been reported [41]. These inflorescences, with completely developed spikelets and terminal florets, were used as axenic explants for callus induction and plant regeneration. This highly efficient procedure for the development of organ-specific differentiating tissues provides a vehicle for genetic transformation using microprojectile bombardment in switchgrass. *In vitro-*grown mature florets also provide an aseptic source of anthers for the production of haploids, and open up the possibilities for *in vitro* fertilization techniques to enhance breeding experiments between ecotypes that are naturally difficult to cross.

### Genetic engineering

**Figure 3:**
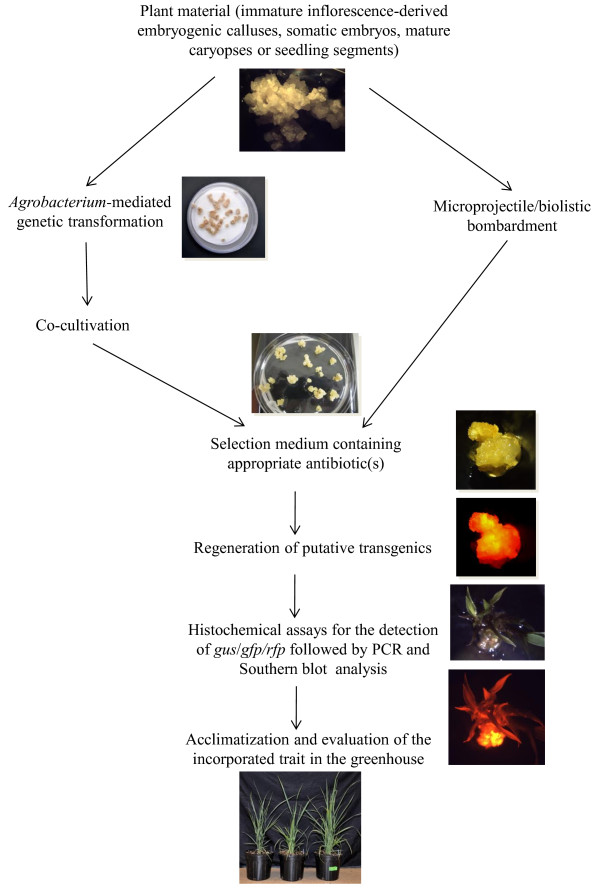
Flow chart of transgenic production in switchgrass [Photo credits: Wegi A. Wuddineh and M Nageswara-Rao].

Genetic transformation is useful for gene discovery and characterization in plant biology. The commercial use of transformation is to introduce traits into plants that would not be possible by conventional breeding alone and also to increase trait development rate [42]. The main trait targets to address using genetic engineering in switchgrass include domestication, plant architecture, and especially reduced recalcitrance for cell wall conversion into biofuel and valuable bioproducts [6,43]. The recent focus on the use of switchgrass as a biofuel crop has led to its large-scale production and genetic engineering (Table 1; Figure 3) for incorporating traits by overexpressing exotic genes and knocking down the expression of endogenous genes [44]. These genes may be for increasing the saccharification efficiency, modifying the cell wall structure and/or composition, enhancing biomass yields or affecting the growth and development of switchgrass plants [6,9,44,45].

**Table 1:** Summary of genetic transformation of switchgrass

**Cultivar used**	**Explant used**	**Method used**	**Gene(s) introduced**	**Reference**
‘Alamo’	Embryogenic calli	Particle bombardment	*sgfp, bar*	[46]
‘Alamo’	Embryogenic calli, somatic embryos, mature caryopses, seedling segments	*At*-mediated, strain AGL1	*uidA, bar*	[47]
‘Alamo’, ‘Alamo 2’	Protoplasts	PEG-mediated	*GUS*	[54]
‘Alamo’	Embryogenic calli	*At*-mediated, strain AGL1	*bar, phaA, phaB, phaC*	[62]
‘Alamo’	Harvested leaves	Agroinfiltration using *At* strain C58C1	*uidA*	[52]
‘Alamo’	Embryogenic calli	*At*-mediated, strain EHA105	*hpt, gusA, GUSPlus*	[49]
‘Alamo’	Germinating seedlings	*At*-mediated, strain AGL1	*GUSPlus*	[53]
‘Alamo’, ‘Performer’, ‘Colony’	Embryogenic calli	*At*-mediated, strain EHA105	*hpt, sgfp*	[50]
‘Alamo’	Embryogenic calli	*At*-mediated, strain AGL1	*Cg1*	[80]
‘Alamo’	Embryogenic calli	*At*-mediated, strain EHA105	*COMT*	[68]
‘ALBA4’, ‘ALBA22’	Embryogenic calli	*At*-mediated, strain AGL1	*hpt, PviCAD2*	[73]
‘HR8’	Embryogenic calli	*At*-mediated, Strain C58C1	*Pv4CL1*	[67]
‘Alamo’	Embryogenic calli	*At*-mediated, strain AGL1	*hpt, Pre-OsmiR156b*	[81]
‘Alamo 2’, ‘ST1’	Embryogenic calli	*At*-mediated	*bar, hpt, GUSPlus, pporRFP*	[61]
‘ST2’	Embryogenic calli	*At*-mediated	*PvMYB4*	[74]
‘Alamo’, ‘Cave-in-Rock’	Dehusked and husked seeds	*At*-mediated, strain EHA105	*npt*II, *gusA, bar, hpt*	[51]

The first transgenic switchgrass was obtained through bombardment of immature inflorescence-derived embryogenic calluses of ‘Alamo’ using a dual marker plasmid comprising the reporter gene *sgfp* (green fluorescent protein; GFP) driven by the rice actin (*Act1*) promoter and the selectable *bar* gene (conferring tolerance to the herbicide Basta) driven by the maize ubiquitin (*Ubi1*) promoter [46]. The leaf tissues and pollen of transgenic plants exhibited GFP and were also tolerant to Basta. T_1 _seedlings from crosses between transgenic and non-transgenic control plants that inherited the *bar* transgene were also tolerant to Basta [46]. *Agrobacterium tumefaciens*-mediated transformation has been accomplished in switchgrass, and appears to be the most common method for switchgrass transformation. The hypervirulent *A. tumefaciens* strain AGL1 carrying the binary vector pDM805 containing the *bar* gene under the control of the *Ubi1* promoter and the *uidA* gene driven by *Act1* promoter was used for transforming four different explant types of which somatic embryos gave the highest transformation frequency [47]. This opened up new opportunities for genetic manipulation of switchgrass as *Agrobacterium*-mediated transformation is often the preferred method since it favors the integration of a low copy number of transgenes. Somleva et al. [48] was able to influence the transformation efficiency of switchgrass by manipulating explant type and genotype, pre-culture treatment of the explant, wounding of explants preceding infection, addition of acetosyringone during inoculation and cocultivation, and selection. These experiments have been valuable in making switchgrass transformation more routine.

Embryogenic calli derived from caryopses or inflorescences of ‘Alamo’ were transformed using *A. tumefaciens* strain EHA105 in combination with the binary vectors pCAMBIA 1301 (carrying a *gusA* from *E. coli*) and pCAMBIA 1305.2 (carrying a *GUSPlus* from *Staphylococcus* spp.) [49]. Since both binary vectors carried the *hygromycin phosphotransferase* gene (*hpt*) as a selectable marker, the transgenic plants were selected on medium supplemented with hygromycin. T_1 _plants from crosses between transgenic and non-transgenic control plants that had multiple copies exhibited transgene silencing, whereas lines harboring only one insert expressed the transgene [49]. One of the largest sources, if not the largest source of efficiency improvement, has come from genotype. Highly regenerable and transformation-competent embryogenic calli developed from seeds of ‘Alamo’, ‘Performer’ and ‘Colony’ were used for genetic transformation using *A. tumefaciens* strain EHA105 containing the binary vectors pTOK47 (carrying a 20 kb *KpnI* fragment of Ti plasmid from pTiBo542, which contains *virB*, *virC* and *virG* virulence genes) and pJLU13 (a derivative of pCAMBIA 1301 containing *hpt* and *sgfp* genes) [50]. It appears that lines of ‘Performer’ are probably the best switchgrass for tissue culture and transformation. Application of vacuum during infection and dehydration at co-cultivation also enhanced the transformation efficiency, as did resting after infection and before culturing onto the selection medium [50]. Transformation efficiency can be improved by the optimization of the gene delivery system, and the appropriate selection and regeneration of transformed cells. Transformation efficiency was enhanced by utilizing the basal parts of ‘Alamo’ seedlings that had higher regeneration potential [51]. Genetic transformation of the type II callus derived from the inflorescences of switchgrass on LP9 medium [27] exhibited transformation efficiency of as high as 34% and also decreased the time taken for transgenic production by one month [52].

Though a number of procedures are well established for switchgrass plant transformation, evaluation of the transgene expression may take several weeks. To reduce this time required for testing gene constructs, transient transgene expression could be a rapid screen [53]. Inoculation of germinating ‘Alamo’ seedlings using an *Agrobacterium*-mediated transient gene expression system (agroinfiltration) was optimized using AGL1, C58, EHA105, and GV3101 strains, of which AGL1 showed the highest efficiency in gene delivery [54]. In another study, it was reported that EHA105 was more effective in gene delivery than LBA4404 or GV3101 [51]. To study the effects of agroinfiltration conditions such as mechanical wounding (bead beating, sonication or vortexing), concentration of the surfactant (Break-Thru S 240, Silwet L77 or Li700), and application of vacuum on transient β-glucuronidase expression, experiments were performed using harvested switchgrass leaves or seedlings [53,54]. Though bead beating wounded the leaf surface, it did not have any effect on the transient β-glucuronidase expression [53]. On the other hand, utilization of sonication and vortexing with carborundum had a positive effect on the transient expression [54]. Use of ‘Break-Thru S 240’ under low vacuum application improved the transient expression [53] while Silwet L77 or Li700 had a negative effect [54]. Transient expression was also enhanced by increasing the vacuum application when surfactant concentration was low [53]. Incorporation of chemicals (L-cysteine and dithiothreitol), heat stress and separation by centrifugation also influenced transient transgene expression [54]. Agroinfiltration might provide a quick assay for overexpression studies in switchgrass.

Mazarei *et al. *[55] developed a protoplast system using leaves and roots of ‘Alamo’ and the ‘Alamo2’ clone followed by transient expression of polyethylene glycol (PEG) mediated DNA uptake in protoplasts [55]. GUS driven by either the CaMV 35S promoter or the maize *ubi1* promoter was utilized as the reporter gene. To develop a transformation system for upland cultivars, calli were induced from seedling segments of the upland octoploid cultivar ‘Cave-in-Rock.’ However, the callus was not amenable for regeneration and produced only roots [51]. Since the tissue culture and transformation systems have been developed for ‘Alamo’ or its derivatives, for a wide applicability across the species, there is a need to create more genotype-independent methodologies for switchgrass. It is also highly crucial to select the right candidate gene(s) for genetic transformation, and develop appropriate protocols for evaluation of transgenics with the non-transgenics [56]. Given the strong germplasm effects observed, this might be a difficult task. In addition, ‘Alamo’ and ‘Performer’ are both agronomically viable lowland cultivars.

A wide variety of promoters have been used for monocot transformation [57-59], but only a few of these have been utilized in switchgrass [46,47,50]. Thus, attention has been given toward promoter testing and discovery for switchgrass genetic engineering [60,61]. Two novel switchgrass ubiquitin gene (*PvUbi1* and *PvUbi2*) promoters have been tested [60]. Particle bombardment of callus using these two promoters exhibited expression patterns comparable to the maize Ubi1 promoter and much higher than that using the 35S promoter [60].

To rapidly screen transgenes in switchgrass, monocot-effective plant expression vectors are required. One such new vector set is pANIC, which uses a Gateway-compatible cassette for over-expression or RNAi of the target gene [62]. The set contains selectable marker and visible marker cassettes for *Agrobacterium*-mediated transformation as well as biolistic bombardment [62]. These vectors were designed especially for switchgrass and are being routinely used in several switchgrass transformation labs.

### Production of bioproducts in transgenic switchgrass

Somleva *et al. *[63] demonstrated the amenability of transgenic switchgrass to synthesize polyhydroxybutyrate (PHB), a biodegradable polyhydroxyalkanoate biobased plastic, in which the pathway was engineered into switchgrass. PHB was accumulated to 3.72% and 1.23% (dry weight) in the leaves and whole tillers respectively. PHB production was stable in the next plant generation too. This study has shown the incorporation of a complex trait in switchgrass is possible for biomanufacturing.

### Cell wall modification

Genetically modified feedstocks play an important role in scenarios for next-generation biofuel production [64]. Reducing lignin biosynthesis can lead to lower recalcitrance and higher saccharification efficiency, making lignin composition and amount an obvious target to change in lignocellulosic feedstocks [6]. Recalcitrance of cell walls conversion to biofuels is perhaps the greatest hurdle in realizing the economic potential of switchgrass and other lignocellulosic biofuel feedstocks [64,65]. Currently, to enable efficient enzymatic degradation of cellulose, harsh physical or chemical pretreatment is required for the modification of the cell wall structures, removal of lignin and degradation of the hemicelluloses [66]. For augmenting the biofuel production from lignocellulosic feedstocks, changing lignin composition and amount are being performed [67,68].

Fu *et al.* reported a reduction in lignin content, and increase (38%) in ethanol yield from transgenic switchgrass in which the endogenous *caffeic acid O-methyltransferase* (*COMT*) gene was down-regulated [69]. The syringyl:guaiacyl monolignol ratio was decreased and the transgenic plants required less pretreatment and enzymes to yield the same levels of ethanol using simultaneous saccharification and fermentation. As a result, there was also enhanced forage quality in the *COMT* down-regulated lines.

The last step in the biosynthesis of lignins is catalyzed by cinnamyl alcohol dehydrogenase (CAD) [70]. CAD deficiency modifies the lignin structure, reduces the lignin content, and augments the saccharification efficiency in grasses [71,72]. *Agrobacterium*-mediated transformation was utilized for RNAi of CAD in switchgrass [73,74]. These two studies reported a reduction in lignin content and increased saccharification efficiency in the transgenic lines. Another important enzyme involved in the biosynthesis of lignin is 4-coumarate:coenzyme A ligase (4CL). Xu *et al.* carried out phylogenetic analysis and gene expression studies, and suggested the involvement of *Pv4CL1* in the biosynthesis of lignins [68]. *Pv4CL1* down-regulated transgenic switchgrass plants, obtained by *Agrobacterium*-mediated transformation, had normal biomass yields with reduced lignin content and increased saccharification efficiency [68].

In contrast to the above-mentioned approach in which endogenous lignin biosynthesis genes were down-regulated, Hui Shen and colleagues targeted the overexpression of a key transcription factor affecting the expression of many lignin biosynthesis genes [75]. A decrease in recalcitrance in transgenic switchgrass was observed when the repressor, *PvMYB4* was overexpressed [75]. The transgenic lines exhibited a drastic reduction in lignin, but no change in the S:G ratio. The plants were also morphologically affected, having more tillers and reduced height. The transgenics had increased cellulose and pectin contents, significantly reduced wall recalcitrance and phenolic fermentation inhibitors, and produced approximately 1.8-fold more ethanol using yeast based simultaneous saccharification and fermentation without pretreatment (Shen *et al.,* in review).

These efforts have highlighted the usefulness of lignin biosynthesis or lignin repressor gene targets for down-regulation, and these genetically engineered plants for reduced lignin may contain higher levels of free monolignols and other phenylpropanoids. The accessibility of cell wall carbohydrates for the production of biofuels is negatively correlated with the amount of lignin present [76,77]. Decrease in lignin content or alteration in its composition alleviated the digestibility of the cellulose and hemicelluloses. This led to enhanced saccharification efficiency, reduction in the severity of the pretreatment, decrease in enzyme requirements and increase in the energy available to microorganisms for breaking down the carbohydrates [69,76,78]. To change the lignin content of the biomass, dwarfing might also be of use as it shifts the biomass allocation from the stem to the leaves [44]. Reduced lignin content during the vegetative phase in switchgrass might also delay flowering, which could also increase vegetative biomass [44,79].

In is unclear whether the lignin biosynthetic pathway is perfectly conserved between widely-studied model species and switchgrass. There might be many more genes and transcription factors that have not been discovered in switchgrass and be manipulated for improved biofuel production. Other cell wall targets include cellulose, reducing the crystallinity of cellulose, hemicellulose, pectin, and their interactions with lignin. Research on the expression of cellulases, *in planta*, under extreme conditions and its thermal stability also needs to be carried out. The cost of lignocellulosic ethanol production may also be reduced by genetically modifying switchgrass to produce the enzymes that are required during fermentation. Devising strategies for recycling these enzymes will also lead to reduction in biofuel production cost.

### Altering switchgrass development: microRNAs and other targets

Improvement in the rate of saccharification efficiency, which is inhibited by the complex structure of the plant cell wall, is an important objective in developing a competent and lucrative biofuel industry [80,81]. Biomass yield could be enhanced by manipulating microRNAs (miRNAs) that regulate transcription factors controlling growth and development in plants [69,81-84]. The maize *Corngrass1* (*Cg1*) gene, which produces a miR156, targets the SQUAMOSA PROMOTER BINDING LIKE (SPL) family and reduces lignification while promoting juvenile characteristics in plants [85,86]. To study how juvenile characters improve the biofuel potential of switchgrass, the *Cg1* gene was constitutively overexpressed in ‘Alamo’ [81]. A second miR156 study overexpressed the switchgrass *PvmiR156* in switchgrass, [82]. In both studies, the transgenic plants had delayed flowering, variant morphology, and improved sugar release. Transgene expression levels were sufficient to allow three morphology categories to be observed. Low expressers resembled non-transgenic switchgrass. Moderate expression levels rendered plants that were shorter and with more tillers. The plants had delayed flowering, which could be useful in bioconfinement of transgenes. High levels of miR156 accumulation induced severe dwarfism and reduced biomass accumulation [81,82]. Thus, targeted overexpression of miR156 could not only make biofuel production more efficient but allow the production of switchgrass that is more suitable for production. These studies highlight the potential utility of this approach for the domestication of new switchgrass cultivars, and the lack or delay in flowering will have important implications for the limitation or prevention of transgene flow into native/wild relatives or non-transgenic agronomic plantings of switchgrass. Recently, it was demonstrated that the expression levels of miR156 and miR162 could be changed under drought conditions in switchgrass [87].

Genetic engineering can also be used to increase the biomass by modifying the plant growth regulators such as increasing the biosynthesis of gibberellins [88] to improve the growth and increase the biomass in switchgrass. Thus, early transgenic research in switchgrass has revealed that multiple targets for improvement have been reached. It appears that there could be a tradeoff between sugar release and plant growth, but results are promising with regards to increasing liters per hectare. To date, there has been no transgene stacking in switchgrass, which should be pursued. For example, it makes sense to hybridize miRI56 plants with those with greatly reduced lignin, such as *MYB4* overexpressers. In addition, tissue-specific and inducible expression of transgenes will also be valuable in decreasing off-target effects. Targeted expression is particularly needed for genes, such as those that are master regulators, to diminish or better control pleiotropic effects. The transgenic studies to date with switchgrass show the power of the technology, which is becoming increasingly routine.

## Genetic and genomics resources

### Molecular markers

A number of DNA marker systems such as restriction fragment length polymorphism (RFLP), chloroplast DNA, randomly amplified polymorphic DNA (RAPD), amplified fragment length polymorphism (AFLP) and simple sequence repeats (SSRs) have been developed for the genetic diversity assessment and phylogenetic studies in switchgrass [20,89-95]. Marker studies helped delineate upland and lowland variation and are useful in developing germplasm conservation and breeding programs [96]. Genetic linkage maps have been constructed using single dose restriction fragments (SDRFs), SSRs, sequence-tagged sites (STS) markers, expressed sequence tags (EST)-derived SSRs, gene-derived STS markers, and diversity array technology (DArT) markers [97-101]. Linkage maps will aid in the identification of quantitative trait loci linked with biomass yield, plant composition and other important agronomic traits, providing a genetic framework to facilitate marker-assisted breeding and genomics research in switchgrass.

Over the last few years, even though various technologies have emerged for whole genome sequencing, it is still technically difficult and expensive to completely sequence complex polyploid species such as switchgrass [102,103]. Transcriptome sequencing of expressed sequence tags (ESTs) is amenable for any organism, including those for which *de novo* whole genome sequencing is difficult, thereby aiding in gene discovery and annotation [103-107]. ESTs have been successfully used for identification of molecular markers, analysis of tissue-specific patterns of expression or for comparative genomics [105,108]. cDNA libraries derived from leaf, stem, crown, and callus of ‘Kanlow’ were utilized for generating 11,990 individual sequences of which 7,810 were unique gene clusters [105]. Sequence similarity and functional classification of these unique gene clusters was also performed. EST sequence information can also be mined for DNA sequence polymorphisms for single nucleotide polymorphisms (SNPs) and SSRs that can be used for genome characterization and genetic diversity assessment [94]. For developing SSR markers, Tobias *et al.* assessed the unique gene clusters and reported the occurrence of short tandem repeats in 3.8% of the ESTs tested [105]. ESTs were also produced by end-sequencing of callus, crown, and seedling tissue derived cDNA libraries of ‘Kanlow,’ and the assembled consensus sequences were aligned with the sorghum genome [108]. They observed that 3.3% of the sequences were similar to potential cell wall related genes. Millions of ESTs from tissue or xylem cell-specific EST libraries of ‘Alamo’ are also now available (http://compbio.dfci.harvard.edu/tgi/cgi-bin/tgi/gimain.pl?gudb=switchgrass) [56].

SSRs and EST-SSRs are significant resources for developing dense linkage maps and identifying economically important traits for utilization in molecular breeding programs intended to develop superior switchgrass cultivars [94]. EST-SSR markers were identified and assessed for the production of fragment length polymorphisms in the two individual parents of a mapping population [108]. To identify SSR sequences longer than 20 bp, available sequence data from switchgrass were assessed using the program SSRIT and approximately 32 genic di-, tri- and tetranucleotide repeat SSRs were characterized [109,110]. When used to differentiate ‘Alamo’ and ‘Kanlow’ individuals, these SSRs exhibited a high degree of polymorphism consistent with their tetraploid, allogamous genome states [110]. Using genomic DNA of ‘SL93 7 × 15’, Wang *et al.* reported the construction of five genomic SSR-enriched libraries and identified 1,300 unique SSR-containing clones [94]. Given the power of genomics as described above, continued expansion of sequence availability, especially when assembled switchgrass genome is made available, will enable better understanding of switchgrass biology as well as facilitate genetic engineering.

### BAC libraries and physical mapping

Efforts to map important traits for enhancing the breeding programs, and utilizing map-based cloning for the isolation of target genes are dependent on the availability of extensive physical and genetic maps; a switchgrass physical map is needed [111]. Genome assembly for switchgrass requires the genome structure information that can be obtained by sequencing bacterial artificial chromosome (BAC) libraries [96,112,113]. ‘Alamo’ has been extensively used in switchgrass breeding programs and is the parent of several mapping populations, therefore, it follows that the current whole-genome sequencing effort is focused on an ‘Alamo’ clone; the clone chosen was termed AP13. AP13 and all Alamo is a heterozygous tetraploid with two subgenomes [96]. Saski *et al.* assembled the first BAC library, by incomplete digestion of nuclear DNA of the ‘Alamo’-derived genotype, SL93 2001–1 with EcoRI, which had approximately ten-fold coverage of the total nuclear content and five-fold of each of the two genomes based on a genome size of 3.2 gigabases (~1.6 Gb per genome) [111]. Since the study was restricted to a single locus and restriction enzyme, it warranted the need of additional libraries to attain fair and near-complete depiction for genome-wide studies. Recently, two (HindIII- and BstYI-fragmented) BAC libraries were constructed from AP13, which also aided in discoveries of SSRs [113]. Comparative analysis with other grass genomes such as foxtail millet, sorghum, rice, maize, and *Brachypodium* revealed high levels of homology with switchgrass exhibiting high microcolinearity with foxtail millet as compared with sorghum [114]. In addition, HudsonAlpha/Joint Genome Institute (JGI) has generated BAC-end sequences from a collection of BACs (http://genomicscience.energy.gov/). These studies provided a precise BAC-based physical platform that offers a definitive approach for sequencing and assembly of the switchgrass genome. They will also be able to give a precise estimate of the GC content, distribution of known, novel and repeat elements, and, thus, of the genome structure and composition of switchgrass.

### Sub-organelle genome sequencing

Chloroplasts are invaluable for genetic and phylogenetic studies. Switchgrass chloroplasts are often maternally inherited and can be transformed, in several other plant species, to deliver high recombinant protein production [115,116]. To differentiate genetic diversity in whole chloroplast genomes and a large number of nuclear loci in switchgrass, a unique strategy utilizing high-throughput sequencing of multiplexed restriction-digested reduced-representation libraries was used for the identification of SNPs [117]. The SNPs identified were able to characterize eight haplogroups. Switchgrass chloroplast genomes were also sequenced from individuals of the upland (‘Summer Lin2’) and lowland (‘Kanlow Lin1’) ecotypes giving an insight regarding the amount of variation within the two ecotypes, and facilitated comparisons within the ecotypes as well as among other sequenced plastid genomes [118]. These studies emphasize the use of chloroplast genome for comparing genetic variation between the upland and lowland ecotypes, are highly desirable for robust phylogenetic studies and can be used in differentiating mixed population into up- or lowland ecotypes. The complete chloroplast genome will facilitate the generation of species-specific transformation vectors [119] and will create an opportunity for the utilization of plastid genetic engineering in switchgrass.

### Whole genome sequencing

Basic characterization of the switchgrass genome indicates that the tetraploid lowland cultivars have a nuclear DNA content of 3.07 ± 0.06 pg per nucleus [120], resulting in an effective genome size of ~1600 Mb for ‘Alamo’ derived genotypes, which is approximately twice that of sorghum and about three and a half times that of rice [111,121]. Even with the availability of new and modern technologies, whole genome sequencing (WGS) of switchgrass would be difficult to achieve due to its large genome size and polyploidy. A practical solution to Sanger sequencing may be provided by pyrosequencing or other such next generation sequencing (NGS) technologies that offer quick and inexpensive technologies for transcriptomics by avoiding extensive and comparatively low throughput steps [122-124]. For *de novo* sequencing and transcriptomics of complex genomes, 454 pyrosequencing is the most extensively exploited NGS technology. GS FLX Titanium, the latest 454-sequencing platform, can produce a typical read length of approximately 330–700 bases [125,126].

‘Alamo’ AP13 has been chosen for WGS by JGI (http://genome.jgi.doe.gov/genome-projects/). Sequencing of AP13 cDNA libraries produced from various tissues of switchgrass utilizing GS-FLX Titanium technology produced large number of reads for *de novo* assembly, and EST and SSR identification [103]. The accessibility to the foxtail millet draft genome also enhanced the switchgrass EST assembly and nearly doubled the EST information in the public domain. JGI also used a combination of Roche 454-based and Illumina-based sequencing to produce the switchgrass genomic sequence [96]. Initial investigations on assembly of switchgrass genome onto the foxtail millet framework led to the identification of paralogous assemblies from homoeologous assemblies [114]. However, autonomous assembly of both the subgenomes to achieve chromosome-scale contiguity for the reference is challenging [96]. Although dihaploid lines may simplify sequence assembly in switchgrass, they are not preferred for whole genome sequencing because of their elevated infertility and instability [126,127]. The draft genome sequence of switchgrass is now available (http://www.phytozome.net/panicumvirgatum). The genome of switchgrass will help the biologists to determine the function and biotechnological potential of genes, especially those responsible for increasing the biofuel potential such as biomass yield, decreased lignin content and improved saccharification efficiency. Furthermore, comparative analysis of switchgrass with other sequenced grass genomes such as foxtail millet and sorghum will enable a more detailed annotation, and will play an important role in understanding how gene networks evolved and function (National Plant Genome Initiative:2009–2013; http://www.nsf.gov/bio/pubs/reports/). WGS also helps plant breeding efforts.

### Gene expression studies

Information on the fundamental biology and the regulatory mechanisms of gene expression in switchgrass under abiotic stress conditions are required for determining the consequences of genetic improvements and for detection and manipulation of stress tolerance related gene candidates [87]. An Affymetrix microarray chip for switchgrass has been produced that contains representatives of most of its expressed genes that has been used to make a gene expression atlas (http://genomicscience.energy.gov/) [128] as well as the switchgrass relative *Panicum hallii*[129].

Of particular interest with regards to gene expression are miRNA studies. Mature miRNAs inhibit gene expression at the post-transcriptional levels by either targeting mRNAs for degradation or inhibiting protein translation [83,130], which in turn can lead to transcriptional regulatory changes. Switchgrass traits of interest include cellulose biosynthesis, sucrose and fat metabolism, signal transduction, and plant development [131]. Investigations on the effect of salt and drought stress on the expression of miRNAs revealed an altered expression pattern of miRNAs in a dose-dependent manner [87]. Transgenic plants expressing the miR156 gene that exhibited severe morphological alterations was used to investigate the effects of miR156 over-expression on its downstream genes using Affymetrix microarray analysis [82]. The study discovered that transcript abundance reduced in eight SPL gene probe sets, leading to the expression analysis of the corresponding cDNA sequences, which showed that the highest miR156 expressers had the most reduction in *Pv*SPLs transcript abundance [82]. Such gene expression analyses will further augment the characterization and expression of genes controlling the biofuel traits, enhance the functional genomics studies and molecular breeding, and may further help in the assembly of the switchgrass genome.

### Reverse genetics

To take advantage of the new DNA sequence information and to investigate the functions of specific genes, targeting induced local lesions in genomes (TILLING) was developed. TILLING is a non-transgenic technology that utilizes a reverse genetics approach for the production and detection of mutation [132]. EcoTILLING is a variation of TILLING that investigates the natural variation among cultivar/inbred line/accession when aligned with a sequenced reference genome for the identification of SNPs [133,134]. In switchgrass, TILLING, EcoTILLING, or a permutation of both are being utilized [134]. This will lead to the identification of multiple SNPs within a target region of switchgrass accessions and when compared to a reference genome will be able to define the relatedness and differences among the target region. Traits such as biomass yield, saccharification efficiency, and flowering time may be potentially identified in switchgrass using these techniques. The limitations being that the mutations may be introduced randomly throughout the genome, and a large number of individuals need to be screened to identify the mutants having the trait of interest. It will also be difficult to identify recessive mutants due to the polyploidy nature of switchgrass.

## Discussion

Switchgrass has been the topic of important discoveries and relevance in genomics and biotechnology in the last decade [27,46,47,63]. Significant trait improvement via biotechnology e.g. [69,73,74] with increased transformation efficiency [50] has been demonstrated in switchgrass. This suggests that genetic improvements of biofuel properties of switchgrass through expression and down-regulation of transgenes is a practical way to rapidly establish it as a viable bioenergy crop on a commercial level and will be achieved with growing reliability in the coming years [60,81,82]. Though transgenic approaches are considered imperative for the development of switchgrass and other biofuel crops, their cost-effectiveness will be dependent on their domestication, productivity and biofuel properties [44]. However, we speculate that a regulatory necessity, at least in the US, will likely be bioconfinement of transgenes [10,135,136].

Transgene escape has been considered as a major environmental, ecological and regulatory concern. Hence, for commercialization of transgenic switchgrass, efficient and reliable transgene bioconfinement strategies would be enabling, especially in US the geographic center of diversity of switchgrass. While transgenes can be vectored in pollen or seed and less commonly asexually, the prospective for long-distance pollination has made pollen-dispersed transgenes a major concern [137]. One strategy to control gene flow in switchgrass would be to introduce male sterility using transgene-encoded ribonucleases that inhibit pollen formation [138,139]. With switchgrass being wind-pollinated, the excision of transgenes from the pollen genomes using site-specific recombination systems will also be desirable [140,141]. Another strategy would be to use plastid (chloroplasts or mitochondria) transformation for the introduction of cytoplasmic male sterility into switchgrass, and thus developing plastid transformation for switchgrass would be helpful. Since the pollen of most plant species contain no chloroplasts, pollen spread will not introduce the foreign genes into wild or non-transgenic switchgrass populations [142,143]. Thus, strategies for transgene bioconfinement and alleviation of gene flow and research that facilitates the utilization of information and proper regulatory guidelines for transgenic feedstocks are essential in developing the biofuel industry’s infrastructure [10], including that for switchgrass [136]. The challenge is to generate efficient methods and procedures to accomplish elevated levels of agricultural productivity while conserving the environment and natural resources [7].

Recent advances in switchgrass genomics will further facilitate biotechnological interventions as well as its germplasm improvements via conventional and molecular breeding. The close colinearity of the switchgrass genome with other grasses will aid in the elucidation of gene function, regulation, and expression by leveraging off other resources. The application of new knowledge and tools developed from genomic resources such as identification of genes like those involved in the lignin pathway, saccharification efficiency, biomass yield, nutritional quality, and pest resistance will help geneticists and plant genetic improvement managers to overcome the limitations associated with conventional breeding, make sexual hybridization more efficient and manipulate various traits effectively. It is important to keep in mind, however, that the utility of new genetic combinations must be demonstrated ultimately by field trials and the value to consumers.

## Conclusions

The development of switchgrass as a biofuel crop has the potential to contribute significantly to lignocellulosic ethanol production without competing with food and feed crops. Biotechnological advances made to genetically modify important biofuel related traits in switchgrass will play a key role in shaping the future of the switchgrass biofuel industry. Genomic information being generated for switchgrass will further enhance the breeding and biotechnological endeavors. Although plant biotechnology will play an important role to the successful generation of energy crops, it should be followed up with breeding programs aimed at sustaining or improving the significant agronomic attributes which made these plants imperative for biofuel generation to start with, namely resistance to abiotic and biotic factors, and low fertilization requirements [144]. The critical issue to be dealt with is how to improve the conversion efficiency from the solar energy to biofuel energy such that biofuels can meet anthropogenic energy consumption demands and be able to replace the fossil fuels.

## Abbreviations

AFLP: Amplified fragment length polymorphism; BAC: Bacterial artificial chromosome; BAP: 6-Benzylaminopurine; BES: BAC-end sequence; CAD: Cinnamyl alcohol dehydrogenase; Cg1: *Corngrass1*; 4CL: 4-Coumarate,coenzyme A ligase; COMT: *Caffeic acid O-methyltransferase*; 2,4-D: 2,4-Dichlorophenoxyacetic acid; DArT: Diversity array technology; EST: Expressed sequence tags; MS: Murashige and Skoog; NGS: Next generation sequencing; RAPD: Randomly amplified polymorphic DNA; RFLP: Restriction fragment length polymorphism; SDRFs: Single dose restriction fragments; SNPs: Single nucleotide polymorphisms; SPL: SQUAMOSA PROMOTER BINDING LIKE; SSRs: Simple sequence repeats; STS: Sequence-tagged sites; TDZ: Thidiazuron; TILLING: Targeting induced local lesions in genomes; WGS: Whole genome sequencing.

## Competing interests

The authors declare that they have no competing interests.

## Authors’ contributions

MNR and JRS conceptualized, researched, wrote the manuscript and made the figures. CK and CNS conceptualized and critically revised the manuscript. All authors read and approved the final manuscript.
